# Understanding food behavior through territorial identity: evidence on locavorism, certification awareness, and novel food acceptance

**DOI:** 10.3389/fpsyg.2026.1882335

**Published:** 2026-08-27

**Authors:** Fabio Tuscano, Irene Sandi, Pericle Raverta, Jatziri Mota-Gutierrez, Paola Toschi, Claudio Forte, Barbara Loera

**Affiliations:** 1 Department of Veterinary Sciences, University of Turin, Turin, Grugliasco, Italy; 2 Department of Psychology, University of Turin, Turin, Italy

**Keywords:** food certification awareness, food neophobia, food preferences, local food consumption, locavorism, sustainable food systems, territorial identity

## Abstract

Global agri-food systems face increasing challenges related to sustainability, public health, and food security, requiring a transition toward more integrated and demand-oriented approaches. In this context, consumer behavior plays a key role, as food choices are shaped not only by economic factors but also by social and psychological dimensions, including identity. This study examines how configurations of territorial identity encompassing local, national, and global attachments are associated with food-related dispositions and behaviors. Using survey data from 1,237 consumers living in a metropolitan area in Northern Italy, a typology of territorial identity profiles was developed. Results show that locally oriented identities are associated with a stronger attachment to local food and higher food neophobia, whereas more globally oriented profiles display greater openness to novelty. However, behavioral outcomes vary: the purchase of certified food products is mainly driven by knowledge, while local food consumption reflects identity-based orientations. Overall, the findings highlight the ambivalent role of territorial identity in shaping sustainable and innovative food choices.

## 1 Introduction

Global agri-food systems are currently facing unprecedented challenges related to environmental sustainability, public health, food security, and socio-economic resilience (Dammak et al., 2025). Increasing pressure on natural resources, climate change, biodiversity loss, and evolving dietary patterns have intensified the need to transition toward more sustainable food production and consumption models (Moretti et al., 2025). In response, international institutions and policymakers emphasize agri-food systems transformation as key to achieving sustainable development goals, highlighting links between production, supply chains, and consumption. While early debates focused on technological innovation and production-side efficiency, growing evidence suggests that systemic change requires addressing the demand side (Bajželj et al., 2014). Consumers play a pivotal role in shaping market dynamics through their everyday food choices, which collectively influence production strategies, distribution systems, and sustainability outcomes. Consequently, understanding these determinants is essential for the transition toward sustainable, safe, and healthy agri-food systems.

Understanding consumer behavior is a cornerstone for shaping the future of agri-food systems, particularly regarding sustainability, safety, and health (Jia et al., 2024). Food choices are not only driven by economic or sensory considerations, but also by psychological and social factors shaping how individuals perceive and engage with food products (Fernqvist et al., 2024). Among these factors, social identity has emerged as a powerful lens through which food-related attitudes and behaviors can be interpreted (Bartels and Reinders, 2010).

Social identity theory posits that individuals define themselves through their membership in social groups, and that these group affiliations influence values, preferences, and decision-making processes (Tajfel and Turner, 2004). In the food domain, territorial identity orientations (local, national, or global) influence purchase behavior, product evaluation, and openness to alternative food systems (Kim and Huang, 2021). Previous research highlighted how local or national identities may strengthen preferences for local products, while cosmopolitan profiles are linked with greater openness to novelty and diversity in food consumption (Vida et al., 2008).

Food neophobia, defined as the reluctance to try unfamiliar foods, is a key psychological determinant of dietary behavior and openness to innovation (Pliner and Hobden, 1992). Higher levels of neophobia are associated with preferences for familiar, culturally embedded foods and reliance on known and socially validated dietary practices when facing uncertainty or novelty (Siegrist and Hartmann, 2020). Territorial identity orientations may shape this process: strong local or national belonging can reinforce attachment to traditional food practices, while global belonging may encourage openness to diversity and experimentation. Thus, food neophobia reveals how identity-related attachments translate into acceptance or resistance toward changing food environments.

Closely related to identity belonging is consumers' orientation toward local food systems, or “locavorism.” Beyond utilitarian choice, locavorism embodies values linked to territorial belonging, support for local economies, and perceived authenticity. Preferences for local foods act as symbolic expressions of community affiliation and cultural heritage, linking consumption practices to social identity. Locally oriented identities reinforce positive attitudes toward domestic production and short supply chains, whereas cosmopolitan orientations may favor diversified and globally sourced food consumption (Reich et al., 2018).

A complementary perspective is provided by awareness of food certifications, reflecting consumers' understanding of food quality and sustainability labels. Certifications communicate information about origin, production standards, and authenticity, helping consumers navigate complex food markets. However, their effectiveness depends on consumers' awareness and trust in them. Identity orientations may shape certification interpretation: territorially anchored consumers may view origin labels as markers of authenticity and belonging, while globally oriented consumers may associate them with broader guarantees of quality and sustainability (Bravo et al., 2024).

Taken together, these dimensions illustrate how territorial identification influences food-related behaviors, psychological dispositions, value-driven preferences, and informational processes. Their interaction helps explain consumer heterogeneity and informs identity-sensitive strategies supporting more sustainable and resilient agri-food systems.

Despite growing interest in identity-based approaches, the agri-food literature has often examined consumer identities in isolation, focusing on single dimensions such as local identity (Aprile et al., 2016) or ethnocentrism (Bryła, 2019). However, consumer groups rarely possess a single, fixed and common identity. Rather, they navigate multiple and sometimes overlapping spheres of belonging that may coexist and interact in shaping food-related attitudes. To the best of our knowledge, understanding of how combinations of territorial identities influence food behavior remains limited, particularly regarding psychological dimensions, including psychological and emotional aspects (e.g., neophobia and awareness of certified food products), with scarce evidence in regional contexts. As a result, it remains unclear whether different configurations of territorial attachment are associated with distinct patterns of food-related dispositions and behaviors. Consumer identities should therefore be conceptualized as configurations of multiple territorial attachments rather than mutually exclusive categories. Consumers may simultaneously identify with local, national, and global spheres, and these combinations can produce distinct and sometimes contradictory food-related attitudes. A configurational perspective may therefore capture forms of territorial belonging that are overlooked when identities are examined separately. Ignoring this complexity may lead to communication and policy strategies that resonate with some consumers while alienating others or prove ineffective overall.

To fill this gap, drawing on empirical evidence from a quota-based consumer survey conducted in Northern Italy, this study examines whether different configurations of territorial identity are associated with distinct food-related dispositions, namely locavorism and food neophobia, and with food-related behaviors, including engagement with certified food products, local food consumption, and openness to novel foods. Together, these dimensions capture psychological predispositions, value-based orientations, and cognitive engagement with food information, offering a multidimensional perspective on how territorial identity shapes food-related decision-making. The findings are expected to contribute to a more multifaceted understanding of how multiple forms of territorial belonging shape contemporary food consumption.

## 2 Method

### 2.1 Procedure and participants

Data were collected in December 2024 through an online survey administered by KKien, a professional market research institute, using an online consumer panel in Northern Italy. The survey was conducted using the Computer Assisted Web Interviewing (CAWI) method. The study adhered to the ethical standards of the Declaration of Helsinki and was approved by the Bioethics Committee of the University of Turin (Approval No. 0548934).

Quota sampling by age (18–24, 25–34, 35–44, 45–54, 55–65), gender and education levels (low, medium, high), was used to approximate the sociodemographic structure of the working-age population of major metropolitan areas in Northern Italy. The distribution of the study sample across the main sociodemographic characteristics, together with the corresponding official population statistics (Istituto Nazionale di Statistica, 2024) used for quota sampling, is reported in Table A1.

The sample included 1,237 individuals, 50.1% men. Age distribution was as follows: 8.6% were 18–24 years old, 19.8% were 25–34, 19.2% were 35–44, 26.3% were 45–54, and 26.1% were 55–65. Regarding education, 32.1% reported low, 48.3% medium, and 19.6% high educational attainment. Most participants followed an omnivorous diet (89.2%), consistent with the dietary profile of the general Italian population. More than half of the participants were married or cohabiting (56.6%), while 32.4% were single and 11.0% were separated, divorced, or widowed. Overall, 54.4% reported having children. Most participants (62.6%) were primarily responsible for grocery shopping within their household, whereas 33.4% shared this responsibility with another adult.

### 2.2 Measures

**Figure 1:**
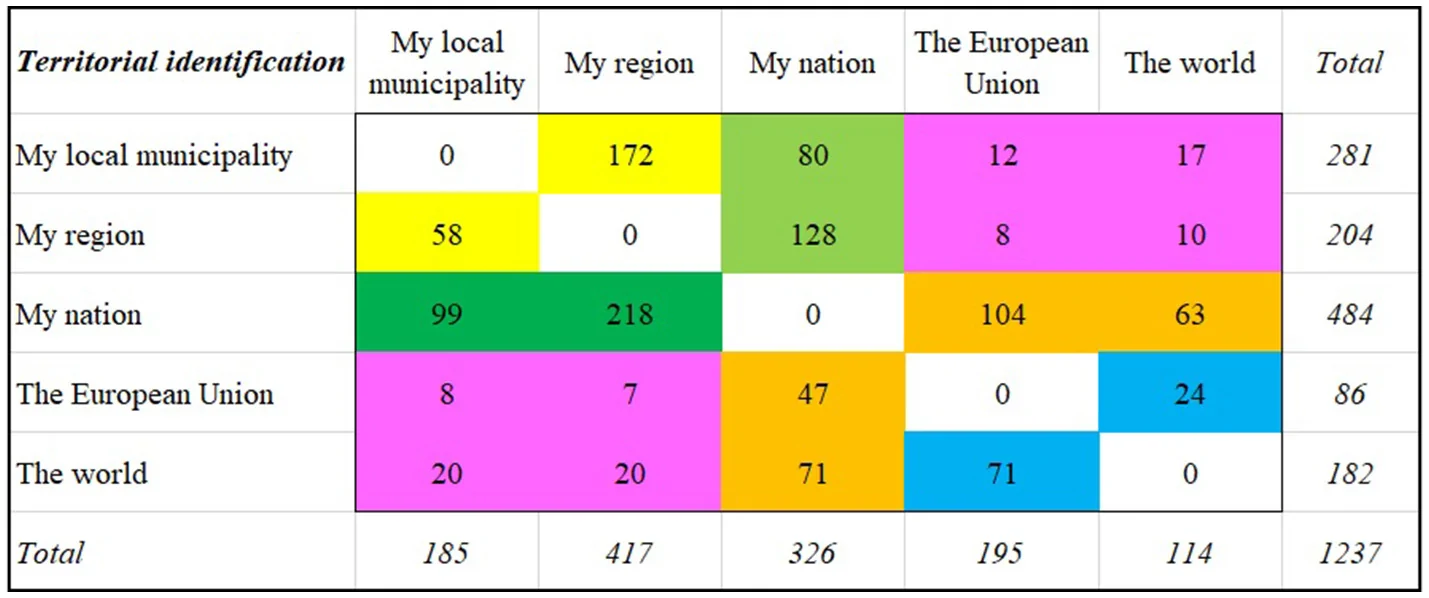
Contingency matrix of primary (row) and secondary (column) territorial identifications, and aggregation procedure used to derive the six territorial identity profiles.

Territorial identity was assessed by asking participants to indicate their primary and secondary territorial belonging (local municipality, region, nation, European Union, or the world). To examine its association with food-related dispositions and behaviors, a typology capturing how multiple and potentially overlapping forms of belonging coexist within individuals was constructed from the cross-classification of responses. This procedure generated a matrix of ordered combinations, grouped into six analytically meaningful profiles reflecting both hierarchical priority and broader orientations toward territorial belonging. In constructing the final categories, similar combinations were aggregated to ensure both theoretical interpretability and adequate group sizes. Figure 1 illustrates the complete contingency matrix and the aggregation procedure used to derive the six territorial identity profiles. Participants who selected both the local municipality and the region, regardless of the order of selection, were classified as local only. Combinations including both local (municipality or region) and national identities were classified according to the order of responses: participants who selected the municipality or region as their primary identification and the nation as their secondary identification were assigned to the local-national profile, whereas those who selected the nation as their primary identification and the municipality or region as their secondary identification were assigned to the national-local profile. This distinction was maintained because the hierarchical priority assigned to local vs. national identification was considered theoretically meaningful and because merging these combinations into a single category would have resulted in an excessively large and heterogeneous group. Combinations including the nation together with either the European Union or the world were classified as national-global; combinations including the local municipality or region together with either the European Union or the world were classified as glocal (global-local); and combinations including both the European Union and the world, regardless of the order of selection, were classified as cosmopolitan.

Locavorism was measured using a seven-item scale adapted from Reich et al. (2018) and the Multilevel Food Ethnocentrism Scale (Ferrales and Grunert, 2024). The scale showed good internal consistency (Cronbach's α = 0.838; McDonald's ω = 0.834), with corrected item-total correlations ranging from 0.522 to 0.655.

Food neophobia was measured with the ten-item by Pliner and Hobden (1992) (Italian version: Guidetti et al., 2018). The scale showed good internal consistency (Cronbach's α = 0.876; McDonald's ω = 0.869). Corrected item-total correlations ranged from 0.486 to 0.712.

For both of these measures, scores were computed by summing responses to the items of each scale. Because the two measures had different ranges, they were standardized (z-scores) to ensure comparability and to avoid scale-related biases in the multivariate analysis of variance (MANOVA).

Knowledge of food certifications was assessed through five dichotomous items referring to certified quality and origin labels (PDO, PGI, PAT, Slow Food Presidium, and Coalvi), adapting measures from Bravo et al. (2024). Responses were summed into a composite index, with higher scores reflecting greater knowledge of food certifications.

Local food consumption was assessed through a single item asking participants whether they regularly purchased local food products or had purchased them in the past (Ferrales and Grunert, 2024). Responses were recoded into a binary variable distinguishing participants who reported regularly purchasing local food products from those who reported only past purchases or no purchases.

Certified food purchase was assessed with a single item asking participants to indicate whether they had purchased or consumed certified food products during the previous 3 months. Responses were recoded into a binary variable distinguishing participants reporting any certified food purchase from those reporting none.

Willingness to try a novel food product combining a traditional local food (agnolotti pasta) filled with an innovative component (cultivated meat derived from cell cultures; “cultivated-meat agnolotti”) was measured on a 10-point response scale, following Loera et al. (2026).

### 2.3 Data analysis

Data were analyzed using IBM SPSS Statistics (version 30.0). Multivariate analysis of variance (MANOVA) was used to examine differences across territorial identity profiles in the two standardized food-related dispositions, locavorism and food neophobia, whereas one-way analysis of variance (ANOVA) assessed differences in knowledge of food certifications. Logistic regression models were then estimated to examine associations between territorial identity profiles, food-related dispositions, and two behavioral outcomes: certified food purchase and local food consumption. Finally, mediation analysis was conducted using PROCESS (Model 4; Hayes, 2022) to examine whether locavorism and food neophobia mediated the association between territorial identity and willingness to try a novel cultivated meat food product. Before estimating the regression-based models, multicollinearity diagnostics were examined. Tolerance values ranged from 0.56 to 0.98 and variance inflation factors (VIFs) from 1.02 to 1.79, indicating no evidence of problematic multicollinearity.

## 3 Results

**Figure 2:**
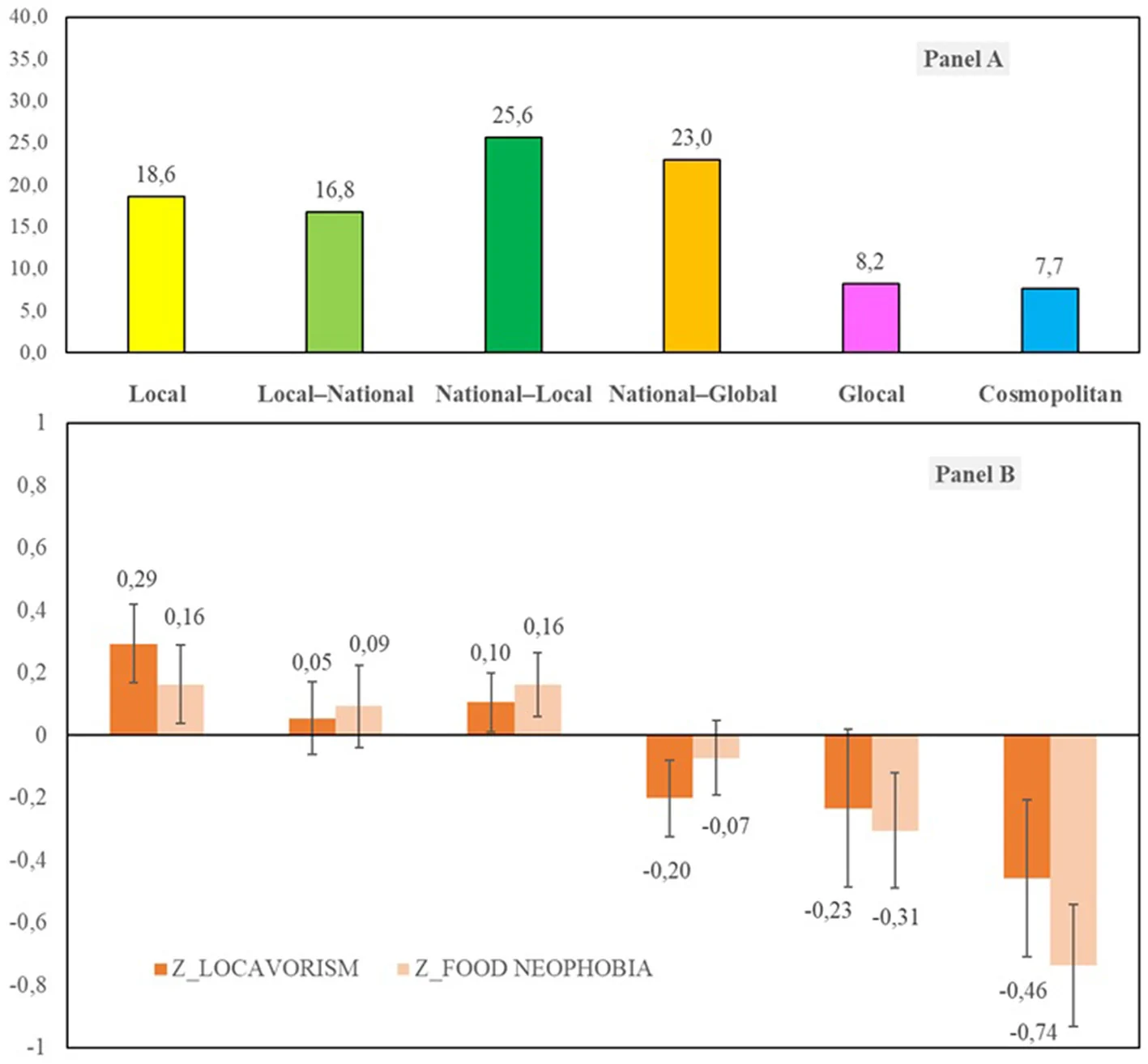
Percentage distribution of territorial identity profiles **(A)** and their associations with food-related dispositions **(B)**: mean standardized scores for locavorism and food neophobia across the six territorial identity profiles, with 95% confidence intervals.

Figure 2, Panel A, shows the six territorial identity profiles derived from the combination of primary and secondary territorial identifications. Participants were distributed as follows: 230 (18.6%) in the local-only profile, 208 (16.8%) in the local-national profile, 317 (25.6%) in the national-local profile, 285 (23.0%) in the national-global profile, 102 (8.2%) in the glocal (global-local) profile, and 95 (7.7%) in the cosmopolitan profile.

The distribution of identity profiles was examined in relation to sociodemographic characteristics. Contingency table analyses revealed a significant association with age and education (both *p* < 0.001), with more globally oriented profiles concentrated among younger and more educated participants, although effect sizes were modest (Cramer's *V* = 0.097 and 0.121, respectively).

Subsequently, the typology was validated by its expected associations with food-related dispositions such as locavorism and food neophobia, as well as with varying levels of knowledge of food certification schemes linked to territorial origin. Consistent with expectations, MANOVA revealed a significant effect of identity profile on the dependent variables, Pillai's Trace = 0.093, F(10, 2462) = 12.04, *p* < 0.001, η^2^*p* = 0.047. Follow-up univariate analyses showed that identity profile significantly affected both locavorism, F(5, 1231) = 13.26, *p* < 0.001, η^2^*p* = 0.051, and food neophobia, F(5, 1231) = 13.67, *p* < 0.001, η^2^p = 0.053.

Inspection of group means (Figure 2B) revealed a clear gradient: more locally oriented identities were associated with higher levels of both locavorism and food neophobia, whereas more globally oriented profiles, particularly cosmopolitan, showed the lowest levels. This pattern indicates that territorial identification systematically structures food-related dispositions, with locally rooted identities associated with stronger attachment to local food and greater reluctance toward unfamiliar foods, and more global identities associated with greater openness.

A one-way analysis of variance (ANOVA) also revealed a significant effect of identity profile on knowledge of food certifications, F(5, 1231) = 2.79, *p* = 0.016, η^2^ = 0.011.Given the violation of the homogeneity of variances assumption (Levene's test *p* = 0.010), *post hoc* comparisons were conducted using the Tamhane correction. Pairwise differences were modest and not statistically significant. This pattern suggests that the significant omnibus ANOVA reflects modest overall variation across identity profiles rather than pronounced differences between specific groups, consistent with the absence of significant pairwise comparisons.Non-etheless, mean scores suggest a slight tendency for individuals with a purely local identity to report lower levels of knowledge, whereas more integrated profiles, particularly glocal orientations, show somewhat higher levels.

After validating the typology, logistic regression models were estimated to examine how territorial identification and food-related dispositions relate to two behavioral outcomes: certified food purchase and local food consumption (Table 1).

**Table 1:** Logistic regression models predicting certified food purchase and local food consumption.

Predictor	Certified food purchase OR (95% CI)	Local food consumption OR (95% CI)
Women	0.55 (0.40–0.75)^***^	0.76 (0.59–0.96)^*^
Age	1.00 (0.99–1.02)	1.02 (1.01–1.03)^***^
Degree	1.39 (0.88–2.18)	1.92 (1.33–2.76)^**^
Diploma	1.53 (1.09–2.16)^*^	1.40 (1.06–1.85)^*^
Knowledge of certifications	1.91 (1.68–2.17)^***^	1.23 (1.11–1.36)^***^
Locavorism	1.04 (1.01–1.07)^*^	1.10 (1.07–1.13)^***^
Food neophobia	0.97 (0.95–0.99)^**^	0.98 (0.96–1.00)^*^
Local–national	1.49 (0.86–2.55)	0.68 (0.45–1.03)
National–local	0.90 (0.58–1.40)	0.65 (0.45–0.94)^*^
Glocal	0.56 (0.30–1.06)	0.58 (0.34–1.01)
National–global	1.47 (0.90–2.40)	0.86 (0.58–1.28)
Cosmopolitan	0.78 (0.40–1.53)	0.47 (0.27–0.81)^**^
Nagelkerke *R*^2^	0.227	0.158
−2 Log Likelihood	1043.55	1509.68
Overall classification	83.8%	67.0%

Values are odds ratios (OR) with 95% confidence intervals (CI). Nagelkerke *R*^2^, −2 log likelihood (−2LL), and overall correct classification are reported as model fit indices. Reference category for territorial identification is Local only; reference categories for education and gender are Low education and Men, respectively. ^*^p < 0.05, ^**^p < 0.01, ^***^p < 0.001.

Certified food purchase was primarily driven by knowledge of certification schemes (OR = 1.91, *p* < 0.001). Locavorism also showed a positive association (OR = 1.04, *p* < 0.05), while food neophobia was negatively associated (OR = 0.97, *p* < 0.01). Among sociodemographic variables, being a woman was associated with lower odds of purchase (OR = 0.55, *p* < 0.001), while holding a high school diploma was associated with higher odds of purchase (OR = 1.53, *p* = 0.014). Territorial identification did not show significant effects, suggesting that certified food consumption is weakly related to identity profiles once knowledge and psychological dispositions are taken into account.

A different pattern emerged for local food consumption. While knowledge of certifications and locavorism remained significant predictors, territorial identity played a stronger role: individuals with a national-local identity (OR = 0.60, *p* < 0.01) and particularly cosmopolitan individuals (OR = 0.48, *p* < 0.01) were significantly less likely to consume local food compared to those with exclusively local identification. This pattern suggests that local food consumption is more strongly embedded in territorial orientations and symbolic attachment to place.

Overall, the comparison of the two models highlights a shift from a knowledge-driven pattern in certified food purchase to a more identity- and value- driven pattern in local food consumption. While engagement with certification schemes appears to depend primarily on cognitive familiarity, local food practices are more closely tied to territorial belonging and identity-related psychological dispositions.

These patterns suggest that territorial identification may be linked to behavior through psychological dispositions. To test this, a parallel mediation model (PROCESS, Model 4) was estimated, with territorial identification treated as a multicategorical predictor, locavorism and food neophobia as mediators, and willingness to try a novel cultivated-meat food product (a cultivated-meat filled pasta, i.e. “agnolotti”) as the dependent variable. Table 2 reports the results of the mediation analysis.

**Table 2:** Parallel mediation model linking territorial identification to willingness to try a novel cultivated-meat food through locavorism and food neophobia.

Territorial identity	Total effect (c) β	Indirect via locavorism	95% Bootstrap CI	Indirect via neophobia	95% Bootstrap CI	Direct effect (c') β
Local–national	0.197	0.059^*^	0.007, 0.132	0.067	−0.064, 0.207	0.071
National–local	−0.178	0.051^*^	0.005, 0.118	−0.022	−0.149, 0.104	−0.207
National–global	0.834^**^	0.112^*^	0.019, 0.221	0.143^*^	0.015, 0.281	0.579^*^
Glocal	0.679	0.103^*^	0.016, 0.219	0.265^***^	0.093, 0.446	0.311
Cosmopolitan	1.854^***^	0.176^*^	0.031, 0.350	0.583^***^	0.376, 0.815	1.095^**^
**Mediators**	β	**SE**	***t***			
Locavorism (*R*^2^ 0.097)	−0.225^*^	0.087	−2.58			
Food neophobia (*R*^2^ 0.093)	−0.758^***^	0.087	−8.67			
**Covariates**	β	**SE**	***t***			
Women	−0.481^**^	0.168	−2.87			
Age	−0.054^***^	0.007	−7.72			
Degree	−0.136	0.253	−0.54			
Diploma	−0.503^*^	0.242	−2.08			
Knowledge	0.327	0.350	0.93			
**Variable**	**Description**		**Percentage sample (%)**		**Percentage population (%)**	
Age (years)	18–24		8.6		11.5	
	25–34		19.8		17.9	
	35–44		19.2		19.1	
	45–54		26.3		24.8	
	55–65		26.1		26.7	
Gender	Men		50.1		50.3	
	Women		49.9		49.7	
Education level	Low		32.1		33.1	
	Medium		48.3		49.0	
	High		19.6		17.9	

All coefficients are unstandardized. Locavorism and food neophobia are standardized variables (*z*-scores). Indirect effects are reported together with their 95% percentile bootstrap confidence intervals based on 5,000 bootstrap resamples. Indirect effects are considered statistically significant when the 95% bootstrap confidence interval does not include zero. ^*^p < 0.05, ^**^p < 0.01, ^***^p < 0.001. Reference category for territorial identification is Local only, for education is Low, for gender is Men.

Results showed that food neophobia represents the main pathway linking territorial identification to willingness to try the novel food. This interpretation was supported by bootstrap confidence intervals, which excluded zero for all significant indirect effects.

Indirect effects through neophobia were larger than those through locavorism and reached statistical significance for more globally oriented identity profiles. In particular, effects via neophobia were strongest for cosmopolitan (β = 0.583, *p* < 0.001) and glocal individuals (β = 0.265, *p* < 0.001), whereas effects via locavorism were smaller (e.g., β = 0.176 and β = 0.103, respectively). National-global and cosmopolitan profiles showed positive indirect effects through both mediators, reflecting lower locavorism and especially lower food neophobia, which in turn were associated with greater willingness to try cultivated meat.

Total effects were significant for the national-global (β = 0.834, *p* < 0.01) and cosmopolitan (β = 1.854, *p* < 0.001) profiles. After accounting for the indirect pathways, direct effects remained significant for these same profiles (national-global: β = 0.579, *p* < 0.05; cosmopolitan: β = 1.095, *p* < 0.01), indicating that territorial identification is associated with willingness to try beyond the psychological mechanisms captured by locavorism and food neophobia. This pattern is consistent with partial mediation.

Both locavorism (β = −0.225, *p* < 0.05) and food neophobia (β = −0.758, *p* < 0.001) were negatively associated with willingness to try cultivated meat, with neophobia showing a substantially stronger effect.

Overall, these findings indicate that differences in openness to novel food are primarily driven by variation in food neophobia across identity profiles, with locavorism playing a secondary role.

## 4 Discussion

This study examined how territorial identification is associated with dispositions and behaviors across three domains: engagement with certified food products, consumption of local food, and openness to a novel food product. Overall, the findings indicate that territorial identification is closely linked to food-related orientations, although its association with consumption behaviors varies across domains.

A first insight concerns the role of knowledge and attitudes. Engagement with certified food products appears to be primarily driven by familiarity with certification schemes, suggesting a largely cognitive mode of engagement in which knowledge plays a central role (Grunert, 2005), while territorial identification plays a more limited role. Although the omnibus analysis indicated significant differences in certification knowledge across territorial identity profiles, *post hoc* comparisons did not identify significant pairwise contrasts. This pattern suggests modest overall variation across identity profiles rather than marked differences between specific groups.

By contrast, local food consumption is more strongly embedded in dispositional and identity-related dimensions. Locavorism emerges as a central correlate of local food consumption, and behavioral patterns differ across territorial identity profiles, with more globally oriented profiles being less likely to consume local food. This highlights that local food practices are tied not only to preferences but also to broader orientations toward place and belonging.

A different pattern emerges for novel food acceptance. Willingness to try a novel cultivated-meat food is primarily associated with openness to novelty, with food neophobia as the strongest predictor. Locavorism is also negatively associated, suggesting that attachment to local and traditional food practices may limit openness to innovation. In this domain, knowledge of certification schemes is not significant, further suggesting that openness to food innovation depends more on psychological dispositions than on cognitive familiarity with food systems.

The mediation analysis indicated that the association between territorial identification and openness to novel food is largely accounted for by differences in food neophobia, and only to a lesser extent by locavorism, in line with Proi et al. (2025). More globally oriented identities were associated with lower food neophobia and weaker locavorist orientations, both of which were associated with greater willingness to try cultivated meat. At the same time, residual direct effects suggest that territorial identification captures broader symbolic orientations not fully explained by these mechanisms.

Within this framework, the findings point to a potential “dark side” of locavorism and local identity. While locally oriented identities are often viewed as beneficial for supporting local economies and sustainability, the results provide only partial support for this assumption. Although local attachment is associated with local food consumption, it is also linked to higher food neophobia and lower openness to innovation, and does not consistently translate into stronger engagement across domains.

In this sense, local identity may reflect a form of cultural conservatism associated with both place-based consumption and lower openness to new food technologies. This ambivalence aligns with critiques of localism (Born and Purcell, 2006) and research identifying food neophobia as a barrier to novel food acceptance (Pliner and Hobden, 1992; Siegrist and Hartmann, 2020; Yu et al., 2025).

Several limitations of the present study should be acknowledged. First, given the cross-sectional design, the observed mediation effects do not allow confirmation of the temporal sequence required for causal interpretations. Second, local food consumption and the purchase of certified products were assessed using single-item measures. Although single-item measures are commonly used in population-based studies, they may provide lower precision than multi-item scales. Third, willingness to try cultivated meat is a multifactorial outcome that may also be influenced by factors not included in the present analyses, such as political orientation, environmental concern, familiarity with food technologies, and prior meat consumption. Therefore, the possibility of residual confounding cannot be excluded.

Overall, the study shows that territorial identification is a meaningful dimension structuring food-related orientations across domains, highlighting both the enabling and constraining aspects of local attachment in contemporary food systems. More broadly, the findings suggest that considering configurations of territorial belonging, rather than single identity orientations, may provide a more nuanced understanding of food consumption behaviors.

## Data Availability

The raw data supporting the conclusions of this article will be made available by the authors upon reasonable request, without undue reservation.

## Appendix

**Table A1:** Study sample distribution and official population statistics used for quota sampling.

Variable	Description	Percentage sample (%)	Percentage population (%)
Age (years)	18–24	8.6	11.5
	25–34	19.8	17.9
	35–44	19.2	19.1
	45–54	26.3	24.8
	55–65	26.1	26.7
Gender	Men	50.1	50.3
	Women	49.9	49.7
Education level	Low	32.1	33.1
	Medium	48.3	49.0
	High	19.6	17.9

Istituto Nazionale di Statistica (2024).

## References

[B1] Aprile M. C. Caputo V. Nayga R. M. (2016). Consumers' preferences and attitudes toward local food products. J. Food Prod. Mark. 22, 19–42. doi: 10.1080/10454446.2014.949990

[B2] Bajželj B. Richards K. S. Allwood J. M. Smith P. Dennis J. S. Curmi E. . (2014). Importance of food-demand management for climate mitigation. Nat. Clim. Change 4, 924–9. doi: 10.1038/nclimate2353

[B3] Bartels J. Reinders M. J. (2010). Social identification, social representations, and consumer innovativeness in an organic food context: a cross-national comparison. Food Qual. Pref. 21, 347–52. doi: 10.1016/j.foodqual.2009.08.016

[B4] Born B. Purcell M. (2006). Avoiding the local trap: scale and food systems in planning research. J. Plan. Educ. Res. 26, 195–207. doi: 10.1177/0739456X06291389

[B5] Bravo I. Colamatteo I. Balzano S. Cappelli L. Iannucci E. (2024). Consumer behaviour regarding certified food. Sustainability 16:3757. doi: 10.3390/su16093757

[B6] Bryła P. (2019). Regional ethnocentrism on the food market as a pattern of sustainable consumption. Sustainability 11:6408. doi: 10.3390/su11226408

[B7] Dammak K. Zouari A. Sidhom L. (2025). Integrating multi capital approaches for enhancing sustainability in agri-food supply chains. Discov. Sustain. 6:1229. doi: 10.1007/s43621-025-02028-5

[B8] Fernqvist F. Spendrup S. Tellström R. (2024). Understanding food choice: a systematic review of reviews. Heliyon 10:e32492. doi: 10.1016/j.heliyon.2024.e32492 38952383 PMC11215270

[B9] Ferrales C. Grunert K. G. (2024). Multilevel food ethnocentrism: cross-national scale development. Food Qual. Pref. 118:105164. doi: 10.1016/j.foodqual.2024.105164

[B10] Grunert K. G. (2005). Food quality and safety: consumer perception and demand. Europ. Rev. Agric. Econ. 32, 369–91. doi: 10.1093/eurrag/jbi011

[B11] Guidetti M. Carraro L. Cavazza N. Roccato M. (2018). Validation of the revised Food Neophobia Scale (FNS-R) in the Italian context. Appetite 128, 95–9. doi: 10.1016/j.appet.2018.06.004 29883684

[B12] Hayes A. F. (2022). Introduction to Mediation, Moderation, and Conditional Process Analysis: a Regression-Based Approach, 3rd edn. New York, NY: The Guilford Press.

[B13] Istituto Nazionale di Statistica (2024). Popolazione Residente Per Sesso, età e Stato Civile al 1° *Gennaio 2024. Demografia in cifre*. Roma: ISTAT. Available online at: https://demo.istat.it/app/?i=POSanda=2024andl=em (Accessed July 9, 2026).

[B14] Jia F. Shahzadi G. Bourlakis M. John A. (2024). Promoting resilient and sustainable food systems: a systematic literature review on short food supply chains. J. Clean. Prod. 435:140364. doi: 10.1016/j.jclepro.2023.140364

[B15] Kim S.-H. Huang R. (2021). Understanding local food consumption from an ideological perspective: locavorism, authenticity, pride, and willingness to visit. J. Retail. Consum. Serv. 58:102330. doi: 10.1016/j.jretconser.2020.102330

[B16] Loera B. Raverta P. Bertero A. Cresti M. Stano S. Lo Sapio L. (2026). Beyond borders: a cross-national study on cultivated meat acceptance in Italy, France, and the Netherlands. Innov. Food Sci. Emerg. Technol. 109:104459. doi: 10.1016/j.ifset.2026.104459

[B17] Moretti E. Loreau M. Benzaquen M. (2025). A global systems perspective on food demand, deforestation and agricultural Sustainability [Preprint]. doi: 10.2139/ssrn.5617712

[B18] Pliner P. Hobden K. (1992). Development of a scale to measure the trait of food neophobia in humans. Appetite 19, 105–20. doi: 10.1016/0195-6663(92)90014-W 1489209

[B19] Proi M. Coderoni S. Perito M. A. (2025). Social identity and intent to try cultured meat: beyond locavorism and cultural openness. Br. Food J. 128, 1641–60. doi: 10.1108/BFJ-08-2025-1097

[B20] Reich B. J. Beck J. T. Price J. (2018). Food as ideology: Measurement and validation of locavorism. J. Consum. Res. 45, 849–68. doi: 10.1093/jcr/ucy027

[B21] Siegrist M. Hartmann C. (2020). Consumer acceptance of novel food technologies. Nat. Food 1, 343–50. doi: 10.1038/s43016-020-0094-x 37128090

[B22] Tajfel H. Turner J. C. (2004). “The social identity theory of intergroup behavior,” in Political Psychology, eds. J. T. Jost and J. Sidanius (Psychology Press) 276–93. doi: 10.4324/9780203505984-16

[B23] Vida I. Dmitrović T. Obadia C. (2008). The role of ethnic affiliation in consumer ethnocentrism. Europ. J. Mark. 42, 327–43. doi: 10.1108/03090560810852968

[B24] Yu Y. Wassmann B. Lanz M. Siegrist M. (2025). Willingness to consume cultured meat: a meta-analysis. Trends Food Sci. Technol. 164:105226. doi: 10.1016/j.tifs.2025.105226

